# pGlyco: a pipeline for the identification of intact N-glycopeptides by using HCD- and CID-MS/MS and MS3

**DOI:** 10.1038/srep25102

**Published:** 2016-05-03

**Authors:** Wen-Feng Zeng, Ming-Qi Liu, Yang Zhang, Jian-Qiang Wu, Pan Fang, Chao Peng, Aiying Nie, Guoquan Yan, Weiqian Cao, Chao Liu, Hao Chi, Rui-Xiang Sun, Catherine C. L. Wong, Si-Min He, Pengyuan Yang

**Affiliations:** 1 Key Lab of Intelligent information Processing of Chinese Academy of Sciences (CAS), Institute of Computing Technology, CAS, Beijing, China; 2 University of Chinese Academy of Sciences, Beijing, China; 3 Institutes of Biomedical Sciences, Fudan University, Shanghai, China; 4 National Center for Protein Science (Shanghai), Institute of Biochemistry and Cell Biology, Shanghai Institutes for Biological Sciences, Chinese Academy of Sciences, Shanghai, China; 5 Thermo Fisher Scientific Co., Ltd, Shanghai, China

## Abstract

Confident characterization of the microheterogeneity of protein glycosylation through identification of intact glycopeptides remains one of the toughest analytical challenges for glycoproteomics. Recently proposed mass spectrometry (MS)-based methods still have some defects such as lack of the false discovery rate (FDR) analysis for the glycan identification and lack of sufficient fragmentation information for the peptide identification. Here we proposed pGlyco, a novel pipeline for the identification of intact glycopeptides by using complementary MS techniques: 1) HCD-MS/MS followed by product-dependent CID-MS/MS was used to provide complementary fragments to identify the glycans, and a novel target-decoy method was developed to estimate the false discovery rate of the glycan identification; 2) data-dependent acquisition of MS3 for some most intense peaks of HCD-MS/MS was used to provide fragments to identify the peptide backbones. By integrating HCD-MS/MS, CID-MS/MS and MS3, intact glycopeptides could be confidently identified. With pGlyco, a standard glycoprotein mixture was analyzed in the Orbitrap Fusion, and 309 non-redundant intact glycopeptides were identified with detailed spectral information of both glycans and peptides.

Confident characterization of the microheterogeneity of protein glycosylation remains one of the toughest analytical challenges^1^,^2^. Interpretation of intact glycopeptides by using liquid chromatography coupled with mass spectrometry (LC-MS) is one of the most promising methods for site-specific glycosylation study so far^3^. Different kinds of MS techniques and corresponding bioinformatic tools have been developed for the interpretation of intact glycopeptides.

One approach is direct interpretation of intact glycopeptides by using CID-MS/MS coupled with ETD-MS/MS or targeted MS3^4^,^5^. Generally, in a CID-MS/MS spectrum, sufficient Y ions could be observed to deduce the glycan of a glycopeptide (In glycoproteomics, a Y ion of a glycopeptide is the peptide backbone ion carrying a glycan fragment from the glycosidic bond cleavage, and a y ion of a glycopeptide is the y ion of its peptide backbone). Some software tools have been developed to identify glycans by CID-MS/MS^5^,^6^,^7^,^8^,^9^. However, the b and y ions of the peptide backbone are usually undetectable in a CID-MS/MS spectrum^4^, so the peptide backbone identification should be performed by using some other MS techniques. One of them is ETD-MS/MS, which has extensive peptide backbone cleavage. By integrating the complementary information of CID- and ETD-MS/MS, intact glycopeptides could be confidently identified^10^. However, the sensitivity and the applicable scope of ETD-MS/MS are arguably limited as compared with HCD- and CID-MS/MS in current generation of MS instruments^11^,^12^,^13^, though some supercharging methods such as TMT tagging have been used to improve the sensitivity of glycopeptide identification in ETD-MS/MS analysis^14^,^15^. Another interesting MS technique for peptide backbone identification is targeted MS3, and the integrated identification pipeline is named as Sweet-Heart^5^, in which theoretical Y_1_ ions are firstly predicted by CID-MS/MS, and then multiple rounds of targeted MS3 are performed based on these Y_1_ ion predictions. Peptide backbones are confirmed after identifying these MS3 spectra.

The other popular method for the identification of intact glycopeptides is HCD-product-dependent-ETD (HCD-pd-ETD), which has been widely adopted in recent years^12^,^16^,^17^. Diagnostic glyco-oxonium ions in HCD-MS/MS spectra could be used to trigger the succeeding ETD dissociation, which could restrict the ETD-MS/MS data acquisition to only true glycopeptide precursors. HCD-MS/MS has additional two advantages for identification of intact glycopeptides: 1) Y_1_ ions are recognizable through fine-tuning the normalized collision energy (NCE)^18^, which could help trigger the MS3 fragmentation of Y_1_ ions easily from an HCD-MS/MS spectrum. And in an HCD-MS/MS spectrum, some Y ions could also be detected for the identification of the glycan^19^,^20^; 2) additional b and y ions of the peptide backbones of some glycopeptides in HCD-MS/MS spectra enable the Y_1_-based peptide search such as “Sweet-Heart for HCD” or MAGIC, which replaces the precursor mass of an HCD-MS/MS spectrum with the mass of the Y_1_ ion, and then the peptide backbone may be identified with a conventional protein identification search engine^12^,^21^. An alternative search strategy for the identification of intact glycopeptides with ETD-MS/MS or HCD-MS/MS is the direct protein database search by considering each glycan as a common variable modification attached on the glycosylation site^12^,^14^,^22^. However, it has been explicitly shown that this strategy would result in a high false-positive rate even if the peptide-spectrum match score is high, because the FDR control is just applied at the peptide level, with no control for the glycan identification^12^.

As discussed above, peptide backbone identification and glycan FDR estimation are two of the most challenging problems in glycoproteomics. To address these two issues, we proposed a new pipeline called pGlyco, which included two new features: 1) complementary fragments from both HCD-MS/MS and CID-MS/MS were used to identify glycans, and a novel target-decoy method was developed to estimate the false discovery rate of the glycan identification; 2) data-dependent acquisition (DDA) of MS3 for some most intense peaks in the HCD-MS/MS spectrum was used to identify peptide backbones. In the HCD-MS/MS spectrum of a glycopeptide, the presence of the Y_1_ ion as one of the most intense ions above 700 m/z allows an MS instrument to perform the MS3 data acquisition of the Y_1_ ion in the data-dependent acquisition mode, resulting in a fully automated MS3 acquisition with no need to predict the prior Y_1_ ion information as in the targeted-MS3. And MS3 spectra of Y_1_ ions could generate sufficient fragments to identify peptide backbones. By combining these two features, intact glycopeptides could be identified with detailed spectral information for both glycans and peptides. We applied pGlyco to the study of a mixture of 6 standard glycoproteins and identified 309 non-redundant intact glycopeptides. pGlyco is currently available for free download at http://pfind.ict.ac.cn/software/pGlyco1505/.

## Methods

### Materials

A uniform mixture of six standard glycoproteins was used as the starting material, including IgG (56834, catalog numeber), IgA (I4036), IgM (I8260), Alpha-1-acid glycoprotein (G9885), Alpha-2-macroglobulin (M6159) and Haptoglobin (H3536) (Sigma). Trypsinization and HILIC enrichment were performed as previously reported^22^.

### LC-MS/MS conditions

The standard glycoprotein mixture was analyzed by nanospray LC-MS/MS on an Orbitrap Fusion Tribrid (Thermo Scientific) coupled to an EASY-nano-LC System (Thermo Scientific) without the trap column. For one LC-MS run, 10 ug mixture was used (before HILIC enrichment). In our experience, for glycoprotein mixtures, about 1 ~ 5% sample remained after HILIC enrichment. Therefore, the loading was about 0.1 ~ 0.5 ug. The mixture was loaded onto a C18 spray tip 15 cm × 75 μm i.d. column and was separated at a flow rate of 350 nL/min by using a gradient of 3% to 22% solvent B (100% acetonitrile with 0.1% formic acid) in 42 min, followed by an increase to 30% B in 6 min, and then to 90% B in 6 min and held for another 6 min. Solvent A was 0.1% formic acid in water.

Two separate LC-MS/MS runs were performed: the first one collected HCD-MS/MS and CID-MS/MS spectra, and the other one collected HCD-MS/MS and MS3 spectra.

### HCD-pd-CID-MS/MS

The parameters used for MS data acquisition of HCD-MS/MS and CID-MS/MS spectra were: (1) MS: top speed mode, cycle time = 3 sec; scan range (m/z) = 400–2,000; resolution = 60,000; AGC target = 200,000; maximum injection time = 5 ms; MS1 precursor selection range = 700–2,000; included charge state = 2–6; dynamic exclusion after n times, n = 1; dynamic exclusion duration = 15 sec; precursor priority = most intense; (2) HCD-MS/MS: isolation mode = quadrupole; isolation window = 2; collision energy = 40%; detector type = orbitrap; resolution = 15,000; AGC target = 100,000; maximum injection time = 35 ms; microscan = 1; product ion table: at least n product ions detected, n = 1; product ion threshold = 30%; product ion table = 138.055 Da (the triggering ion of HCD-pd-CID is the 138.055 Da ion with at least 30% relative intensity); (3) CID-MS/MS: isolation mode = ion trap; isolation window = 2; collision energy = 35%; detector type = orbitrap; resolution = 15,000; AGC target = 100,000; maximum injection time = 35 ms; microscan = 1.

### HCD-pd-MS3

HCD-pd-MS3 was performed in another MS run. The parameters used for data acquisition of MS and HCD-MS/MS were the same as the parameters described above. And the parameters used for MS3 acquisition were: exclusion mass list = 100–700; top N = 3 (three most intense ions in the HCD-MS/MS spectrum were subjected to MS3 analysis respectively); isolation mode = ion trap; isolation window = 2; collision energy = 35%; detector type = ion trap; AGC target = 500,000; maximum injection time = 500 ms; microscan = 1.

## Results and discussion

### Workflow of the identification of glycopeptides

Raw data were converted to Mascot Generic Format (MGF) by pParse (version 2.0.6)^23^. After removal of glyco-oxonium ions (see the section “**Glyco-oxonium ions**” and Table S-1 in the Supporting Information), HCD- and CID-MS/MS spectra were deisotoped and then analyzed by pGlyco against a glycan database that combined two previously reported glycan databases^6^,^24^,^25^: 1) three largest plausible N-glycans, corresponding to high mannose-, hybrid-, and complex-type glycan structures in human serum were compiled into one glycan database, containing 2,012 non-isomorphic glycan structures; 2) the GlycomeDB database (http://www.glycome-db.org) recorded 34,457 glycans, and only N-linked glycans were extracted. After combining these two glycan database and removing redundancies, we obtained 2,860 N-glycan structures. For the analysis of the HCD/CID-MS/MS spectrum pair, the mass tolerance for precursors and fragments were set as ±10 ppm and ±20 ppm respectively.

**Figure 1:**
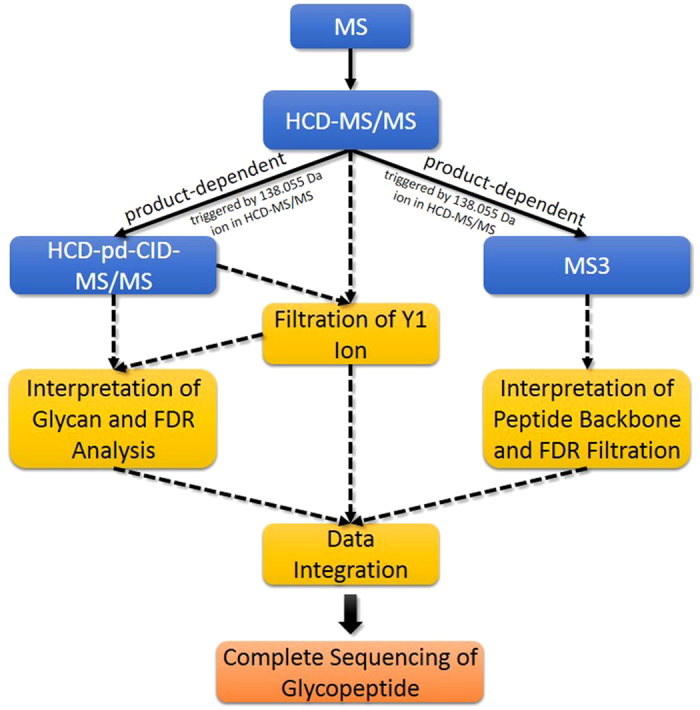
The overall workflow of pGlyco. First the sample is analyzed by HCD-MS/MS (NCE = 40%). Then the product-dependent CID-MS/MS and data-dependent MS3 analyses are performed separately. pGlyco identifies glycopeptides by integrating the distinct information from these three complementary MS acquisitions based on the peptide backbone masses and the retention time. A solid line refers to data acquisition, and a dotted line refers to data interpretation.

pGlyco integrated spectral information from HCD-MS/MS, CID-MS/MS and MS3 to identify glycopeptides. We divided our MS data acquisition and analysis workflow into four steps, as illustrated in Fig. 1:

**Step 1**: HCD-MS/MS.

After full scan, precursors were firstly fragmented by HCD with normalized collision energy (NCE) at 40%. Diagnostic ions in an HCD-MS/MS spectrum were used to determine whether the precursor was a glycopeptide^16^,^17^, and then to trigger the succeeding acquisition of CID-MS/MS or DDA-MS3 spectra only for the true glycopeptides. We found that the peak 138.055 was always the highest peak in a HCD-MS/MS spectrum at 40% NCE, and it was specifically enough to select the true glycopeptide precursors by using 138.055 (see the section “**Glyco-oxonium ions**” in the Supporting Information). Moreover, in HCD-MS/MS at 40% NCE, the Y_1_ ion (the peptide backbone with a HexNAc attached) often coexisted with its corresponding cross-ring fragmentation on the HexNAc residue (i.e., the ^0,2^X_0_ ion)^6^,^21^, which was also considered as one of the trimannosyl core ions in pGlyco. There are 9 trimannosyl core ions used in pGlyco, which are Y_0_ (naked peptide), Y_1_ (peptide + HexNAc_1_), ^0,2^X_0_ (peptide + cross-ring fragment of HexNAc), Y_2_ (peptide + HexNAc_2_), Y_3_ (peptide + HexNAc_2_Hex_1_), Y_4_ (peptide + HexNAc_2_Hex_2_), Y_5_ (peptide + HexNAc_2_Hex_3_), 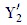 (peptide + HexNAc_1_dHex_1_) and 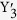 (peptide + HexNAc_2_dHex_1_), as defined in Figure S-1 in the Supporting Information. We named the ^0,2^X_0_ ion as 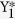 ion for simplicity.

**Step 2**: HCD-pd-CID-MS/MS.

HCD-pd-CID was performed to generate a CID-MS/MS spectrum with the same precursor of the HCD-MS/MS spectrum. For each HCD/CID-MS/MS spectrum pair, the total number of matched trimannosyl core ions was used as a feature to filter the candidate Y_1_ ion of each glycan in the glycan database, and the candidate peptide backbone mass could be deduced. Theoretical Y ions could be calculated as the deduced peptide backbone mass plus the masses of the reducing-terminal fragments of a glycan, and then they were matched and scored against the HCD/CID-MS/MS spectrum pair. A novel target-decoy method with a finite mixture model was used to estimate the false discovery rate (FDR) of the glycan identification.

**Step 3**: HCD-pd-MS3.

Taking the advantage of the novel instrument settings provided by the Orbitrap Fusion, MS3 spectra could be acquired for the three most intense peaks in the HCD-MS/MS spectrum within a certain mass range. And the Y_1_ ion may present as one of the most intense ions in HCD-MS/MS, which enables MS3 acquisition of Y_1_ ions to be performed in a data-dependent mode. MS3 spectra were identified by a protein identification search engine, pFind 2.8, and the conventional target-decoy approach for the peptide identification was employed to estimate the FDR. HCD-pd-MS3 was performed in another MS run to obtain more MS2 and MS3 spectra due to HCD-pd-MS3 need a much longer duty cycle.

**Step 4**: Data integration.

Information of MS/MS and MS3 spectral analysis from the previous three steps was assembled. Glycans identified by HCD- and CID-MS/MS spectrum pairs, and peptides identified by MS3 spectra were aligned based on the peptide backbone masses and the retention time. And then glycopeptides were identified with complete information of both glycans and peptide backbones.

### Filtration of Y_1_ ions

The Y_1_ ion information was a bridge to connect the glycan identification and the peptide backbone identification for intact glycopeptides. For each glycan candidate (in the glycan database) of a given HCD/CID-MS/MS spectrum pair, the Y_1_ ion mass could be deduced by subtracting the glycan mass from the precursor mass of the spectrum pair (Y_1_ ion mass = precursor mass – glycan mass + HexNAc mass), and then the corresponding trimannosyl core ions could be calculated. At the beginning, pGlyco filtered out unreliable candidate Y_1_ ions by the following criteria: there must be at least three trimannosyl core ions matched in the HCD/CID-MS/MS spectrum pair, or the (Y_1_, 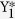) ion pair with the same charge state was matched in the HCD-MS/MS spectrum. After filtration, a mass list of candidate Y_1_ ions were obtained for the spectrum pair, and the peptide backbone mass was deduced from the mass of each candidate Y_1_ ion.

### Interpretation of glycans

For the glycan analysis, both HCD- and CID-MS/MS spectra were used. The scoring scheme for glycan identification of pGlyco was a revised version of the previously reported algorithm for the CID-MS/MS spectral analysis of glycopeptides^6^. With the peptide backbone mass deduced from the mass of each candidate Y_1_ ion, the masses of Y ions resulting from glycosidic bond cleavages were calculated by the mass of the peptide backbone plus the masses of the reducing-terminal fragments of each glycan structure, and then they were matched against the HCD/CID-MS/MS spectrum pair. The scoring scheme of pGlyco considered the matched peaks, their matching mass errors and the number of matched trimannosyl core ions, which was listed below:


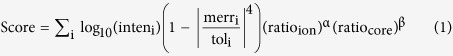


The term inten_i_ is the absolute intensity of a matched peak. The term tol_i_ refers to the matching mass tolerance of fragment ions, e.g. 20 ppm, and merr_i_ refers to the matching mass error ranging from −to l_i_ to +to l_i_. The score of each matched peak is weighted by a quartic polynomial function, 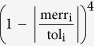, which aims to penalize the larger mass errors with heavier penalties. The term, ratio_ion_, is the ratio of the number of matched ions to the number of theoretical ions, and ratio_core_ is the ratio of the number of matched trimannosyl core ions to the number of theoretical trimannosyl core ions. By cross validation, the parameters α and β were fine-tuned as 0.22 and 0.45 respectively. At last, the top-ranked glycan of each spectrum pair was kept in the final results.

### Interpretation of MS3 data

MS3 data were converted to “.ms3” format by pXtract within pFind Studio (version 2.8)^26^,^27^, and then analyzed by pFind 2.8. The protein database was the database of the six standard glycoproteins mixed with 500 protein sequences randomly selected from SwissProt (v12.05, Homo sapiens species) as the background. Concatenated forward-reverse database search was performed to estimate the peptide FDR. The N-glycosylation sequon (N-X-S/T/C, X ≠ P) was modified by changing “N” to “J” (the two shared the same mass) which had been applied previously^28^,^29^. The enzyme was semi-trypsin and the maximal missed cleavage was 2. Fixed modification was carbamidomethylation on all Cys residues (C + 57.022 Da). Variable modifications contained oxidation on Met (M + 15.995 Da), HexNAc on N-glycosylation sequon (J + 203.079 Da). The matching mass tolerance for precursors of MS3 spectra was set as ± 3 Da. Since MS3 acquisition was performed in the low-resolution ion-trap, the matching mass tolerance for fragment ions was set as  ± 0.5 Da. Neutral mass loss of 203.079 Da of the HexNAc was considered in pFind. Only the peptide-spectrum match (PSM) with the modification of HexNAc on “J” was kept as a valid candidate, which was a potential identity of the Y_1_ ion. And the PSM FDR was set to 1% for these candidate Y_1_ PSMs.

### FDR analysis for glycan identification

After identification of an HCD- and CID-MS/MS spectrum pair, its top-ranked glycan with a deduced peptide backbone mass was output as the glycopeptide candidate, but the error rate was unknown. The FDR estimation for glycan identification has puzzled researchers for a long time^30^. In pGlyco, we developed a novel decoy method coupled with the finite mixture model to give an approximate solution against this problem. For peptide identification, the target-decoy strategy has become a routine method to estimate the FDR, and a sequence-based decoy database generated from the target database is used. However, it is difficult to design a corresponding tree-based target-decoy strategy for glycan identification, so a novel decoy method is to be developed. In pGlyco, we investigated a spectrum-based decoy method to estimate the glycan FDR. For the sequence-based decoy method in peptide identification, each sequence-based decoy peptide is theoretically fragmented into a decoy spectrum, and then matched against the experimental spectrum to get a decoy match. Although the tree-based decoy for a glycan structure is difficult to generate, it is easy to generate a theoretical decoy spectrum to get a decoy match against the experimental glycopeptide spectrum. We named this novel decoy method as the spectrum-based decoy method. When searching an experimental spectrum, the theoretical target glycopeptide spectrum was generated after the masses of the Y ions were deduced based on the putative peptide backbone mass. And then, we added a random mass ranging from 1–30 Da to the mass of each deduced Y ion to generate a theoretical decoy spectrum. Both theoretical target and decoy spectra were competitively matched against the experimental spectrum. The key assumption of target-decoy method is “the number of incorrect identifications from target or decoy sequences are equally likely^31^”. However, this assumption might not always be guaranteed when using the spectrum-based decoy method, and hence the bias of the FDR estimation might arise. To adjust the possible bias, a finite mixture model (FMM) was employed, which had been used for the peptide identification^32^. This method was validated by comparing with the conventional sequence-based target-decoy approach for the peptide identification in public datasets, and showed quite a good performance (see the section “**The spectrum-based decoy method and the FMM**” in the Supporting Information).

**Figure 2:**
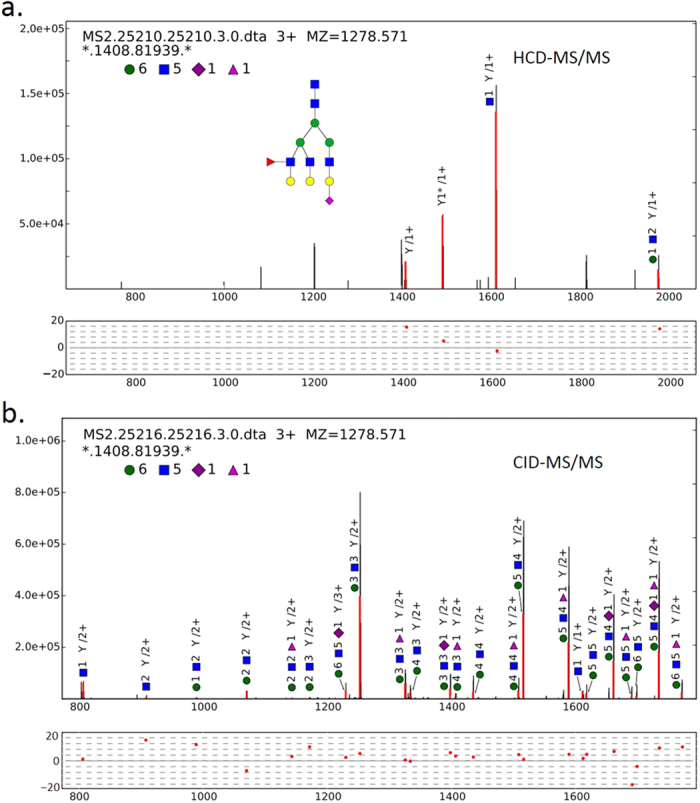
An example of analyzing an HCD- and CID-MS/MS spectrum pair of the glycan (6, 5, 1, 0, 1). (**a**) An example of trimannosyl core ions matched against the HCD-MS/MS spectrum. The deduced peptide backbone mass is 1408.819 Da. In this spectrum, the Y_1_ ion is the most intense ion in the mass range above 700 m/z. (**b**) The sister CID-MS/MS spectrum of the HCD-MS/MS spectrum in (**a**). By combining the HCD- and CID-MS/MS spectrum analysis, the glycan is confidently identified. The tolerance of fragment ions for both HCD- and CID-MS/MS is ±20 ppm.

After employing the spectrum-based decoy and the FMM, distributions of correct and incorrect scores were drawn, and FDR could be estimated (see Fig. S-2 in the Supporting Information). Before glycan FDR filtration, pGlyco obtained 2,704 glycan-spectrum matches (GSMs), and at 1% glycan FDR, 1,720 GSMs were identified. Each GSM was represented by a glycan identified by a HCD/CID-MS/MS spectrum pair together with the deduced mass of the peptide backbone, but the sequence of each peptide backbone remained to be identified. An example of analyzing the HCD- and CID-MS/MS spectrum pair for the glycan (6, 5, 1, 0, 1) was illustrated in Fig. 2. (In this manuscript, a glycan composition is represented by a vector with the form (#Hex, #HexNAc, #NeuAc, #NeuGc, #dHex), the vector (6, 5, 1, 0, 1) means the glycan with composition Hex_6_HexNAc_5_NeuAc_1_dHex_1_.) In Fig. 2a, highly intense trimannosyl core ions matched in the HCD-MS/MS spectrum were observed. With the identified glycan (6, 5, 1, 0, 1), the peptide backbone mass was deduced as 1408.819 Da. From matched ions in the HCD-MS/MS spectrum in Fig. 2a, we could see that the relative intensity of the Y_1_ ion was high enough for the data-dependent acquisition of MS3. Figure 2b showed an example of the annotation of a CID-MS/MS spectrum (the sister spectrum of the HCD-MS/MS spectrum in Fig. 2a). Quite a number of Y ions were matched, and most of the matched fragment mass errors were between −10 and 10 ppm, resulting in a highly confident identification. The most possible glycan structure from our glycan database was also drawn in Fig. 2a.

### Alignment between MS/MS and MS3 identifications

**Figure 3:**
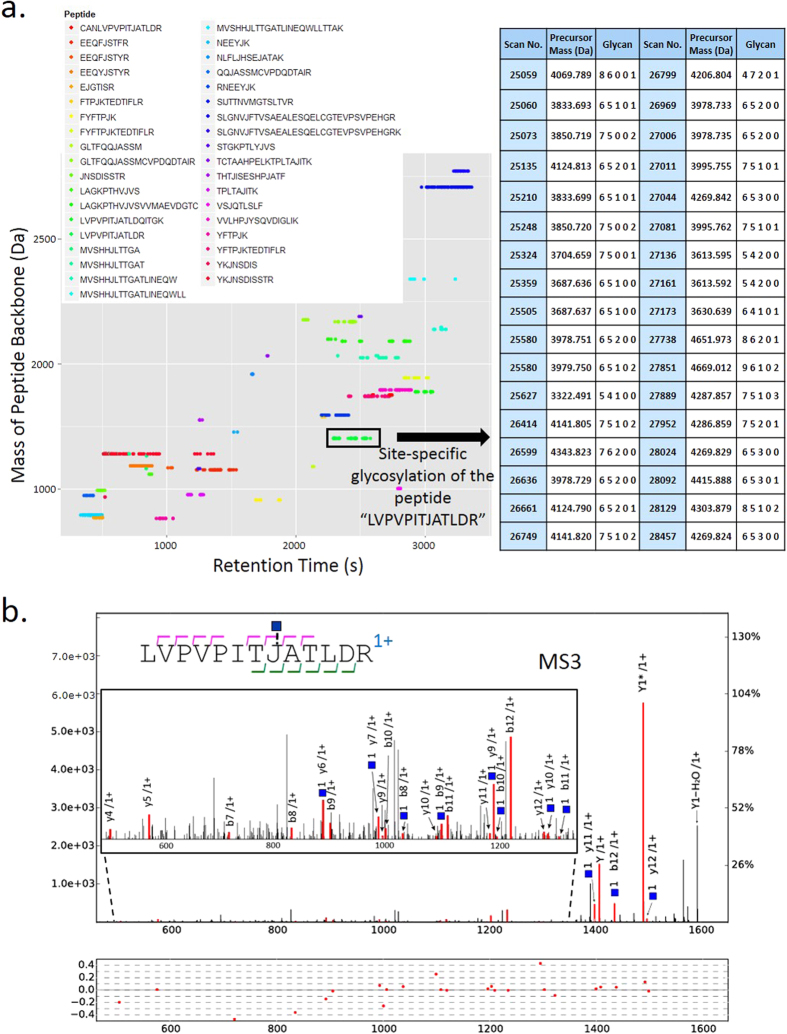
Alignment between the glycans identified by HCD- and CID-MS/MS and the peptide backbones identified by MS3. (**a**) The map of alignment results of glycan identifications and peptide backbone identifications. Different colors of the dots represent the different peptide backbones. The right table shows the microheterogeneity of “LVPVPITJATLDR” characterized by pGlyco. (**b**) The peptide “LVPVPITJ[+HexNAc]ATLDR” identified by MS3. The mass of the peptide “LVPVPITJ[+HexNAc]ATLDR” is 1408.8158 Da, the deduced peptide backbone mass in Fig. 2 is 1408.8194 Da, the precursor mass deviation is 0.0036 Da (2.56 ppm). The tolerance of the fragment ions for MS3 is ±0.5 Da.

Glycans were identified after analyzing the HCD/CID-MS/MS spectrum pairs, but the sequences of peptide backbones were still unknown. In pGlyco, MS3 was used for the identification of peptide sequences of Y_1_ ions. HCD-pd-MS3 was performed in one-hour MS analysis of the standard glycoprotein mixture, and HCD-pd-CID-MS/MS was performed in another one-hour MS run of the same sample. The identified peptide backbones from MS3 analyses and glycans from HCD/CID-MS/MS analyses generated adequate information for the identification of glycopeptides. For MS3 data analyses, 414 PSMs with a HexNAc on “J” (identities of Y_1_) were identified at 1% PSM FDR, and were then compiled into a PSM list for alignment. Alignment between these two MS runs was performed based on the peptide backbone mass and the retention time, as shown in Fig. 3a, where each dot on the map represented a qualified glycopeptide precursor with its peptide backbone mass and retention time as coordinates. Extensive microheterogeneity could be directly perceived from the map: a cluster of horizontally distributed dots represents the microheterogeneity of glycopeptides (identical peptide backbone with different glycans attached). As shown in the map in Fig. 3a, the typical retention time window was 1–6 minutes for a cluster of glycopeptides with the same peptide backbone. Therefore, the peptide backbones identified by MS3 could be aligned with glycans identified by HCD- and CID-MS/MS within a certain retention time window. In the right table of Fig. 3a, a cluster of horizontally distributed dots, corresponding to 34 distinct glycopeptide precursors with the same peptide backbone “LVPVPITJATLDR”, was shown with the information of spectral scan number, the precursor mass and the glycan composition. The match of the peptide backbone “LVPVPITJATLDR” in MS3 analysis was illustrated in Fig. 3b, the peptide backbone was interpreted confidently with nearly twenty matched b/y ions. Combining the results from Figs 2 and 3b, the glycopeptide with the peptide backbone “LVPVPITJATLDR” and the glycan (6,5,1,0,1) was confidently identified.

A PSM list was obtained after MS3 identification at 1% PSM FDR. All identified HCD/CID-MS/MS spectrum pairs before glycan FDR cutoff were then aligned with this PSM list. After alignment, 765 glycopeptide-spectrum matches (GPSMs) were obtained, each GPSM had a glycan identified by a HCD/CID-MS/MS spectrum pair and a peptide backbone identified by a MS3 spectrum. And at 1% glycan FDR, we got 556 confidently identified GPSMs, corresponding to 309 non-redundant glycopeptides. Information including all glycosylation sites and the microheterogeneity was shown in Table S-2. There were 46 potential glycosylation sites (with the sequon N-X-S/T/C, X ≠ P) in our standard protein database, and 25 sites were identified by using pGlyco, but no peptide backbones with the sequon N-X-C were identified, although there were really 3 N-X-C sites in our protein database.

### Feasibility of the spectrum-based decoy method

In pGlyco, the spectrum-based decoy strategy coupled with the finite mixture model was used to estimate the glycan FDR. This novel strategy had been tested in routine peptide identification problems (see Figs S-3 and S-4 in the Supporting Information), and it was still necessary to test if it actually worked in a real glycopeptide dataset.

**Figure 4:**
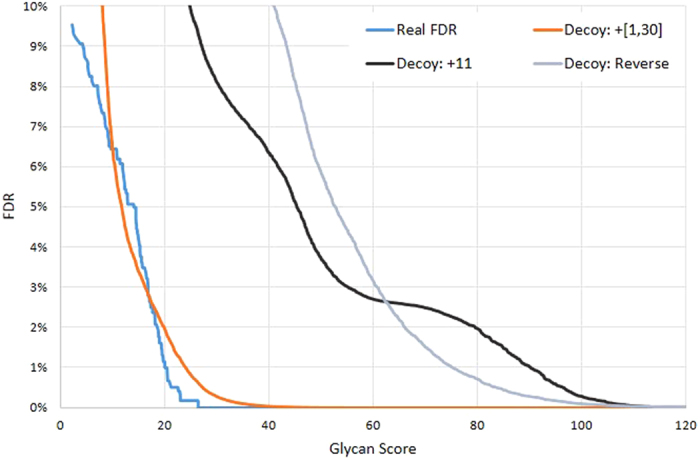
Comparison of different decoy strategies. The estimated FDR of each decoy method is compared with the real FDR. “Decoy: +11” means increasing the mass of each Y ion by 11 Da, and “Decoy: +[1, 30]” means increasing the mass of each Y ion by a random mass ranging from 1–30 Da. “Decoy: Reverse” is simply reversing the Y ions. The finite mixture model is employed for all these decoy methods. “Decoy: +[1, 30]” is the closest estimation to the “Real FDR”.

To further validate the spectrum-based decoy method, we manually checked all the GPSM results. At 1% glycan FDR, 1 out of 556 GPSMs was confirmed as the false identification, while before glycan FDR cutoff, 73 GPSMs out of 765 GPSMs were confirmed as false identifications. The real error rate was calculated based on manually checked results. Although using the 2,704 GSMs to validate the glycan FDR estimation was more rational, it was difficult to judge if a GSM was correct without the identity of the peptide backbone. So here we manually checked the 765 GPSMs, since the peptide backbones were identified at 1% peptide FDR, which meant, when an incorrect GPSM occurred, it was probably because of the incorrect identification of glycan. The real FDR and reported FDR were compared, as shown in Fig. 4. When the reported FDR (the orange curve) was 1%, the real FDR (the cyan curve) was only 0.18%, which showed a conservative estimation by the spectrum-based decoy method. Based on our manually checked results, three spectrum-based decoy strategies were compared: increasing the mass of each Y ion by a fixed 11 Da^30^, or increasing the mass of each Y ion by a random mass ranging from 1 to 30 Da (our decoy method), or reversing the Y ions of each glycan (we defined the reversed Y ion mass = entire glycan mass – Y ion mass), and the results were also shown in Fig. 4. When increasing the Y ion masses by 11 Da as decoys, the FDR was far overestimated. And the FDR was also far overestimated by reversing the Y ions of each glycan structure as decoys, the reason might be that some reversed Y ions of a glycan structure were the same as target Y ions. For example, Figure S-5 in the Supporting Information showed that the Y ions of a glycan structure with the composition (3, 4, 0, 0, 0) were approximately identical to their reversals. Therefore, through our investigation in Fig. 4, Figs S-3 and S-4 in the Supporting Information, our spectrum-based decoy method was proved to be a fine choice to estimate the glycan FDR.

And based on our manually checked data and the novel FDR estimation method, we also investigated the best condition for the Y_1_ ion filtration, as shown in the section “**Best parameter for the Y**_**1**_ **ion filtration**” and Table S-3 in the Supporting Information.

### Complementary ion information provided by HCD- and CID-MS/MS

**Figure 5:**
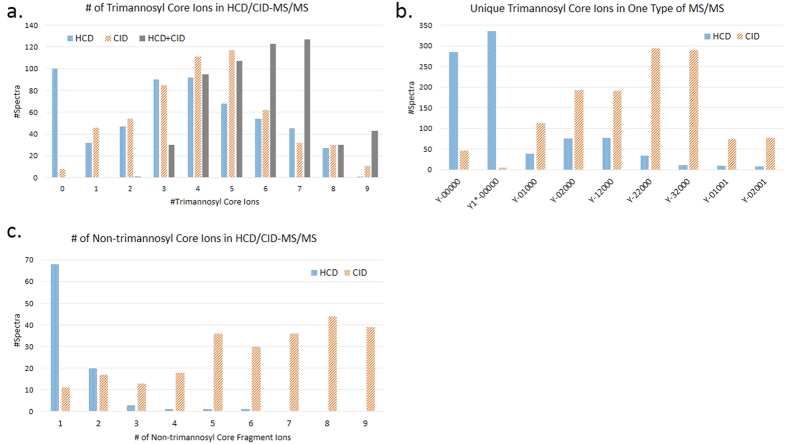
Fragmentation behavior of trimannosyl core and non-trimannosyl core ions in HCD- (**@**NCE = 40%) and CID-MS/MS. (**a**) The number of trimannosyl core ions produced by HCD-MS/MS, or CID-MS/MS, or “HCD + CID” (combination of these two kinds of spectra). (**b**) The number of each trimannosyl core ion uniquely observed in one type of MS/MS. (Y-00000 is the Y_0_ ion and 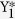-00000 is the 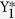 ion. The core-fucosylated ions, Y-01001 and Y-02001, are also counted as trimannosyl core ions). (**c**) The number of non-trimannosyl core ions produced by HCD- and CID-MS/MS. In (**c**) there are 306 CID-MS/MS spectra not shown due to more than 9 non-trimannosyl core ions matched.

Both HCD- and CID-MS/MS could be used to optimize the glycopeptide identification. Recently reported software tools used HCD- or CID-MS/MS to identify glycopeptides, especially the glycan moiety of glycopeptides, but few of them integrated these two fragmentations to further improve the glycopeptide identification^5^,^20^. GlycoFragWork combined HCD- and CID-MS/MS for the glycan identification^11^, but the complementarity should be further investigated. For the glycopeptide identification, trimannosyl core ions, especially the Y_0_, Y_1_, 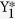 and Y_2_ ions, are critical for detection of the mass of the peptide backbone^12^,^21^. We analyzed the different fragmentation behaviors of trimannosyl core and non-trimannosyl core ions in HCD- and CID-MS/MS spectra, and the results were shown in Fig. 5. More trimannosyl core ions could be provided to improve the glycan identification performance by combining these two fragmentations, as shown in Fig. 5a. And as illustrated in Fig. 5b, HCD-MS/MS (@NCE = 40%) preferentially produced innermost Y ions like Y_0_, 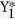 and Y_1_ ions, especially the 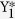 ion, which was almost not observed in CID-MS/MS. The outer trimannosyl core ions such as Y_2_, Y_3_ (Y-12000) and Y_4_ (Y-22000) were more common in CID-MS/MS spectra. From Fig. 5c, we could find out that non-trimannosyl core ions were predominantly produced by CID-MS/MS. For HCD-MS/MS (@NCE = 40%), almost all identified glycopeptides had less than 4 non-trimannosyl core ions matched.

### MS3 fragmentation modes — HCD or CID

We have also investigated the efficiency of different MS3 fragmentation modes for the glycopeptide analysis. In the Orbitrap Fusion, parent ions of MS3 could be fragmented either in the HCD collision cell or in the ion trap (CID). Intuitively, HCD could provide more complete dissociation of precursors than CID. We compared the matched ions of peptides “NEEYJ[ + HexNAc]K” and “LVPVPITJ[ + HexNAc]ATLDR” with close basepeak intensities in HCD- and CID-MS3 spectra, and the results showed that HCD had more peaks matched, implying that HCD was a better fragmentation mode for MS3 identification comparing with CID (see the section “**MS3 fragmentation mode comparison**” and Figs S-6 and S-7 in the Supporting Information).

A potential pitfall of DDA-MS3 for the identification of glycopeptides is the requirement that Y_1_ ion must be one of the three most intense peaks in the mass range above 700 m/z in an HCD-MS/MS spectrum. This requirement is not surely guaranteed for every glycopeptide due to the different fragmentation behaviors of different glycopeptides. Some efforts could be made to get more confident identifications, such as the supplementary targeted MS3 for the unidentified peptide backbones with identified glycans^5^.

## Conclusions

Taking the advantage of advanced settings provided by new instruments, data acquisition for both glycans and peptides becomes more flexible. With the Orbitrap Fusion, MS3 precursors could be acquired for the Y_1_ ions in HCD-MS/MS (@NCE = 40%) in a data-dependent mode. And in HCD (@NCE = 40%) spectra of glycopeptides, 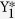 ions are frequently observed, which is counted as one of the trimannosyl core ions in pGlyco to improve the glycan identification. The complementarity of HCD-MS/MS and CID-MS/MS was used to improve the identification performance as well. By integrating the information of HCD-MS/MS, CID-MS/MS and MS3, glycopeptides could be identified with complete spectral information of glycans and peptides.

Another contribution of this work is that we proposed a practical method to estimate the false discovery rate for glycan identifications. By employing the finite mixture model, the score distribution of correct and incorrect identifications could be deconvoluted, and the FDR could be estimated. The method has been tested and proved to work well on two complex protein datasets and a standard protein dataset, and has also been tested on the manually checked glycopeptide data. If any new decoy method is developed, the finite mixture model could be qualified to estimate the FDR as well.

## Additional Information

**How to cite this article**: Zeng, W.-F. *et al*. pGlyco: a pipeline for the identification of intact N-glycopeptides by using HCD- and CID-MS/MS and MS3. *Sci. Rep*. **6**, 25102; doi: 10.1038/srep25102 (2016).

## Supplementary Material

Supplementary Information
