# Supplementary material for: pGlyco: a pipeline for the identification of intact N-glycopeptides by using HCD- and CID-MS/MS and MS3
Source: Sci Rep. 2016 May 3;6:25102. doi: 10.1038/srep25102 (PMC4853738; doi:10.1038/srep25102)
Supplement: Supplementary Information [file srep25102-s1.pdf]

Supporting Information

pGlyco: a pipeline for the identification of intact N-glycopeptides by using HCD- and CID-MS/MS and MS3

Wen-Feng Zeng[1,2,+]; Ming-Qi Liu[3,+]; Yang Zhang[3,+]; Jian-Qiang Wu[1,2,+]; Pan Fang[3]; Chao Peng[4]; Aiying Nie[5]; Guoquan Yan[3]; Weiqian Cao[3]; Chao Liu[1]; Hao Chi[1]; Rui-Xiang Sun[1]; Catherine C. L. Wong[4,*]; Si-Min He[1,2,*]; Pengyuan Yang[3,*]

[1] Key Lab of Intelligent information Processing of Chinese Academy of Sciences (CAS), Institute of Computing Technology, CAS, Beijing, China

[2] University of Chinese Academy of Sciences, Beijing, China

[3] Institutes of Biomedical Sciences, Fudan University, Shanghai, China

[4] National Center for Protein Science (Shanghai), Institute of Biochemistry and Cell Biology, Shanghai Institutes for Biological Sciences, Chinese Academy of Sciences, Shanghai, China

[5] Thermo Fisher Scientific Co., Ltd, Shanghai, China

[*] To whom correspondence should be addressed: P.Y.Y. (pyyang@fudan.edu.cn), S.-M.H. (smhe@ict.ac.cn) and C.C.L.W. (catherine_wong@sibcb.ac.cn)

[+] These authors contributed equally to this work

This document contains the supplementary methods, figures and tables relating to the main text.

References made pertain to this supplementary section only.

Contents

Glyco-oxonium ions 3
The spectrum-based decoy method and the FMM 7
Best parameter for the Y_1 ion filtration 13
MS3 fragmentation modes — HCD or CID 16

Figure S-1 5
Figure S-2 8
Figure S-3 11
Figure S-4 12
Figure S-5 15
Figure S-6 17
Figure S-7 18

Table S-1 4
Table S-2 6
Table S-3 14

Glyco-oxonium ions

Glyco-oxonium ions are frequently present in the spectra of glycopeptides, whose m/z values are: 109.028, 115.039, 126.055, 127.039, 138.055, 144.066, 163.060, 168.066, 186.076, 204.087, 274.092, 290.087, 292.103, 308.098, 366.140, 657.140 and 673.230, and some of them had been reported by [1] (see **Table S-1**). These peaks were removed from all the spectra before identification. Meanwhile, in HCD-MS/MS spectra at 40% NCE, we found that the peak 138.055 was always the most intensive peak for glycopeptides. And our statistics showed that with the peak 138.055 above the relative intensity of 30% in HCD-MS/MS (@NCE = 40%), 99.4% spectra had the 204.087 ion and 93.5% spectra had the 366.140 ion, which implied that it was enough to select glycopeptide precursors by using the ion 138.055. So we used the 138.055 peak to trigger the CID-MS/MS and MS3 for true glycopeptides in our dataset.

Table S-1. Oxonium ions used in pGlyco

Oxonium Ion (m/z)	Marker Type	Formula
109.028	[Hex marker]	$C_6H_4O_2$
115.039	[Hex marker]	$C_5H_6O_3$
126.055	[HexNAc marker]	$C_6H_7O_2N_1$
127.039	Hex−2H_2O	$C_6H_6O_3$
138.055	[HexNAc marker]	$C_7H_7O_2N_1$
144.066	[HexNAc marker]	$C_6H_9O_3N_1$
163.060	Hex	$C_6H_{10}O_5$
168.066	HexNAc−2H_2O	$C_8H_9O_3N_1$
186.076	HexNAc−H_2O	$C_8H_{11}O_4N_1$
204.087	HexNAc	$C_8H_{13}O_5N_1$
274.092	NeuAc−H_2O	$C_{11}H_{15}O_7N_1$
290.087	NeuGc−H_2O	$C_{11}H_{15}O_8N_1$
292.103	NeuAc	$C_{11}H_{17}O_8N_1$
308.098	NeuGc	$C_{11}H_{17}O_9N_1$
366.140	Hex+HexNAc	--
657.140	Hex+HexNAc+NeuAc	--
673.230	Hex+HexNAc+NeuGc	--

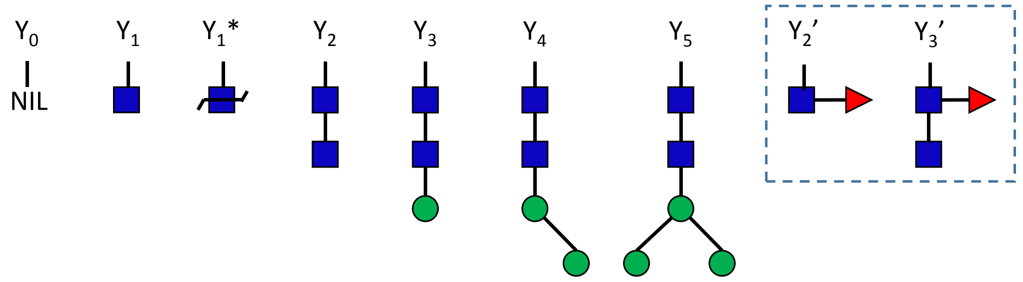

Figure S-1. Trimannosyl core ions used in pGlyco, which are Y_0 (naked peptide), Y_1 (peptide + $HexNAc_1$), Y_1* ($^{0,2}X_0$, peptide + cross-ring fragment of HexNAc), Y_2 (peptide + $HexNAc_2$), Y_3 (peptide + $HexNAc_2Hex_1$), Y_4 (peptide + $HexNAc_2Hex_2$), Y_5 (peptide + $HexNAc_2Hex_3$), Y_2' (peptide + $HexNAc_1dHex_1$) and Y_3' (peptide + $HexNAc_2dHex_1$. Y ions in the dashed box are counted as well for core-fucosylated N-glycopeptides.

Table S-2. Results of site-specific glycosylation study on the mixture of 6 standard glycoproteins

Protein	Accession	Glycosylation Site	Major Peptide(s)	#Glycans	#Spectra
Haptoglobin	P00738	N-241	VVLHPJYSQVDIGLIK	26	38
Haptoglobin / Haptoglobin-related protein	P00738 / P00739	N-184 / N-126	MVSHHJLTTGATLINEQWLLTTAK	15	46
		N-207 / N-149	NLFLJHSEJATAK	1	1
Alpha-2-macroglobulin	P01023	N-410	SIJTTNVMGTSLTVR	17	29
		N-869	SLGNVJFTVSAEALESQELCGTEVPSVPEHGR	11	32
		N-1424	VSJQTLSLF	3	3
Ig gamma-1 chain C region	P01857	N-180	EEQYJSTYR	16	35
Ig gamma-2 chain C region	P01859	N-176	EEQFJSTFR	7	22
Ig gamma-4 chain C region	P01861	N-177	EEQFJSTYR	2	2
Ig mu chain C region	P01871	N-46	YKJNSDISSTR	32	85
		N-209	GLTFQQJASSMCVPDQDTAIR	7	27
		N-272	THTJISESHPJATF	1	1
		N-439	STGKPTLYJVS	3	3
Ig alpha-1 chain C region	P01876	N-340	LAGKPTHVJVSVVMAEVDGTC	9	25
Ig alpha-2 chain C region	P01877	N-205	TPLTAJITK	7	13
Alpha-1-acid glycoprotein 1	P02763	N-33	LVPVPITJATLDQITGK	11	14
		N-103	EJGTISR	18	24
Alpha-1-acid glycoprotein 1 / 2	P02763 / P19652	N-56 / N-56	NEEYJK / RNEEYJK	27	66
		N-72 / N-72	YFTPJKTEDTIFLR	28	52
Alpha-1-acid glycoprotein 2	P19652	N-33	LVPVPITJATLDR	18	38
Total Number	11	25		260	556

The spectrum-based decoy method and the FMM

Scores of decoy identifications could be used to construct the score distribution of incorrect matches, while the score distribution of target identifications is a mixture score distribution of both correct and incorrect identifications. For the target-decoy approach in the field of peptide identification, the basic assumption is "the number of incorrect identifications from target or decoy sequences are equally likely [2]," which might not be surely guaranteed by constructing decoy identifications for the glycan identification. Therefore, to solve this mixture model of target-decoy approach of glycan identifications, we employed a finite mixture model to estimate the density functions of the correct and incorrect score distributions, which were denoted by f(x|+) and f(x|−). And the mixture probability of incorrect identifications, denoted by π_0, was estimated at the same time. f(x|−) was modeled by the finite gamma-mixture model, which used several gamma distributions to fit decoy scores by the expectation-maximization (EM) algorithm, and the number of gamma components was determined by the Bayesian information criterion (BIC) [3]. And f(x|+) was then estimated by finite Gaussian-mixture models. The EM algorithm was also employed, which was listed below:

1. Initialize π_0, the mixture probability of the false positives from f(x|−), and π_{1g}, the mixture probability of each Gaussian component. The mean and variance of each Gaussian component, denoted by u_g and σ_g, are initialized as well. The number of Gaussian components, G, is determined by BIC.
2. E-Step: Estimate the likelihood of the incorrect membership, p_{0i}, and the likelihood of each Gaussian component, p_{gi}, for each target match according to the Bayes rule:

$$p_{0i} = \pi_0 f(x_i|-)/[\pi_0 f(x_i|-) + \sum_{k=1}^{G} \pi_k \mathrm{gauss}(x_i|u_k,\sigma_k)],$$

$$p_{gi} = \pi_g \mathrm{gauss}(x_i|u_g,\sigma_g)/[\pi_0 f(x_i|-) + \sum_{k=1}^{G} \pi_k \mathrm{gauss}(x_i|u_k,\sigma_k)].$$

3. M-Step: Update π_0 by averaging p_{0i}, and π_g by averaging p_{gi}. And u_g and σ_g are re-estimated with the newly updated parameters.
4. Repeat E-Step and M-Step until convergence.

f(x|+) is calculated as the sum of each Gaussian component weighted by corresponding π_{1g}, the two estimated mixture distributions was shown in **Figure S-2** According to the Bayes rule, the PEP can be expressed as PEP(x) = π_0f(x|−)/[π_0f(x|−)+(1−π_0)f(x|+)] given a score x, and at last, the FDR at threshold x can be estimated by the equation: FDR(x) = E[PEP(x)] [4, 5].

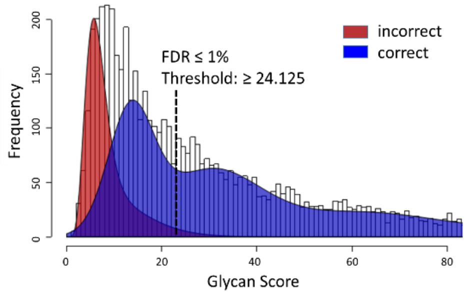

Figure S-2. The false discovery rate analysis is performed by the target-decoy and finite mixture model analysis. Incorrect score distribution (red) is generated from decoy matches. The histogram is generated from the scores of all target identifications. And then the correct distribution (blue) is solved by the finite mixture model from incorrect and target identifications. At 1% FDR, the score threshold is 24.125.

One question about a novel FDR estimation method is that the FDR may be underestimated. As one validation, we tested the finite mixture model (FMM) and the spectrum-based decoy method on routine peptide identification problems; if underestimation occurred in the glycan FDR estimation, it would probably occur in the peptide FDR estimation as well, as a saccharide residue was analogous to an amino acid residue. In our test, two public HCD datasets of *HeLa* cells generated by the Orbitrap Velos and Q-Exactive respectively [6] and an HCD dataset of 8 standard proteins generated by LTQ-Orbitrap XL [7, 8] were used. The RAW data files were converted into MGF files by pXtract, and then were searched by pFind 2.8. The protein sequence database was SwissProt (v12.05, Homo sapiens species) for Velos or QE dataset; for the dataset of standard proteins, the protein sequence database was the sequence of 8 standard proteins mixed with sequences of yeast, as described in [7]. The enzyme was trypsin and the maximal number of missed cleavages was 2. Fixed modifications contained carbamidomethylation on all Cys (C + 57.022 Da). Variable modifications contained oxidation on Met (M + 15.995 Da) and acetylation on the protein N-terminal. The mass tolerance of both precursors and fragments was set as ±20 ppm. In particular, for the glycan identification, pGlyco just used Y ions only, and therefore, to simulate this situation in the peptide identification, we disabled all the b ion matching in pFind 2.8. Concatenated search of forward plus reversed protein sequences was used. Testing results were shown in **Figure S-3**. It showed that the FMM worked well on sequence-based decoy method, the difference between the FDR curves with FMM (cyan) and without FMM (light green) was not too large. And the FDR curve of the spectrum-based decoy method (orange), which was adding a random mass ranging from 1 to 30 Da to each y ion, did not appear to be too much different from the FDR curve of sequence-based decoy method. And the FDR estimated by the spectrum-based decoy method was more conservative than that estimated by the sequence-based decoy method on the dataset of standard proteins, as show in **Figure S-3c**.

The key assumption of the target-decoy method was “the number of incorrect identifications from target or decoy sequences are equally likely [2]”, and we called it the “1:1 assumption”. Based on the “1:1 assumption”, the FDR could be estimated as #decoy/#target. It was widely accepted that the “1:1 assumption” always hold when using the sequence-based decoy method. In the results of Velos dataset, the number of sequence-based decoy identifications was 3515, which implied there were approximate 3515 incorrect results in target identifications. However, the number of spectrum-based decoy identifications was only 1569, the ratio was far from 1:1 (3,515 :

1,569 ≈ 2.25 : 1). And in the QE dataset, the ratio was 9119 to 3691 (9,119 : 3,691 ≈ 2.5 : 1). Therefore, there was no evidence showing that "the number of incorrect identifications from target or decoy sequences are equally likely" or the 1:1 assumption still held when using the spectrum-based decoy method, and this was why we used the FMM algorithm to model the bias of "1:1 assumption", so as to make the FDR estimated by the spectrum-based decoy method be similar to that of the sequence-based target-decoy method.

On the Velos dataset, we also tested the spectrum-based decoy method with different random mass ranges by using the same search parameters, such as adding a fixed mass to each y ion, adding a random mass ranging from −15 to 15 Da, adding a random mass ranging from 1 to 20 or adding a random mass ranging from 1 to 40, and we found that adding a random mass ranging from 1 to 30 Da was at least a sub-optimal spectrum-based decoy method, as illustrated in **Figure S-4**.

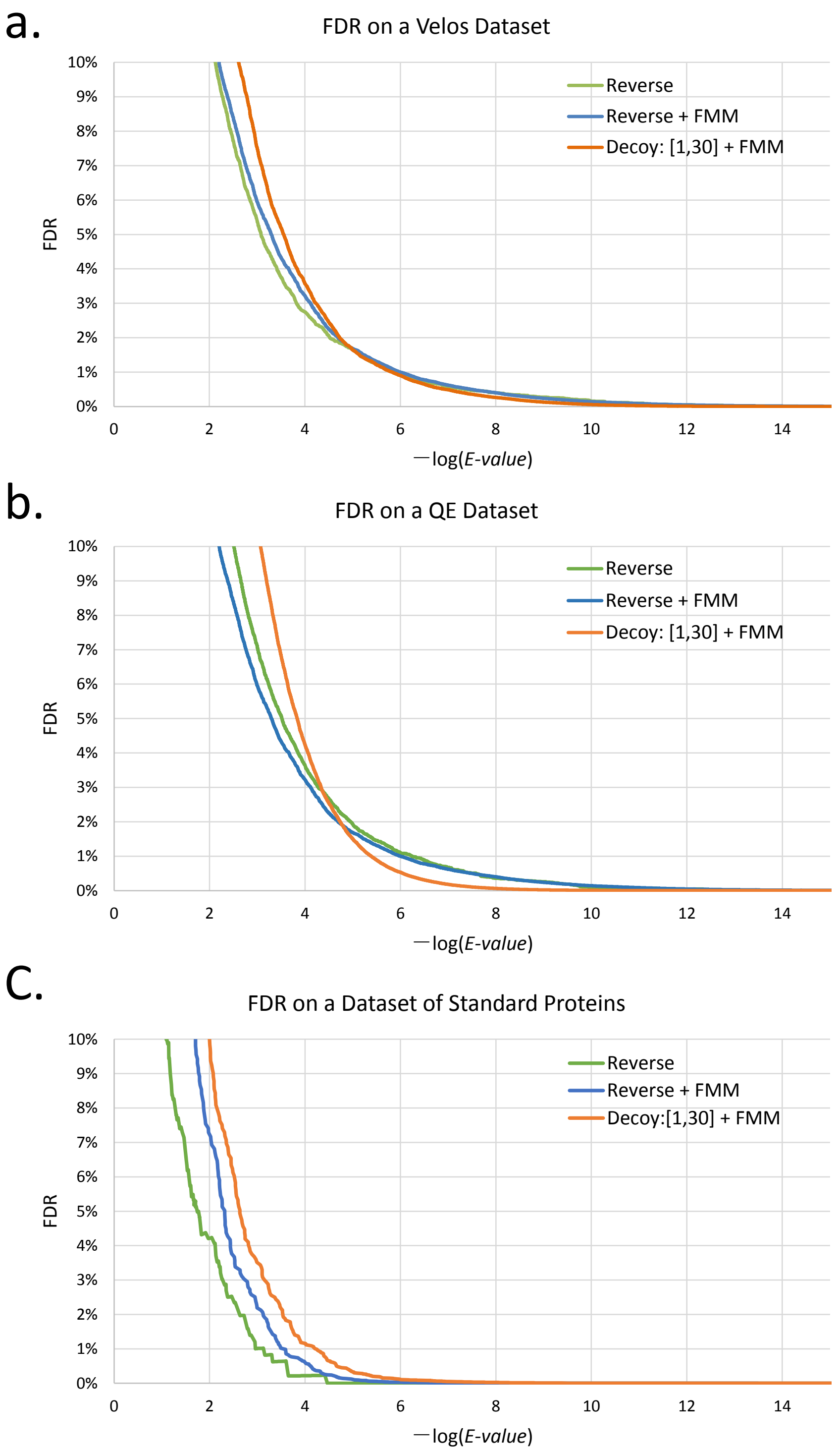

Figure S-3. Testing the finite mixture model (FMM) and the spectrum-based decoy method on three peptide datasets. (a) Testing on an Orbitrap Velos dataset. (b) Testing on a Q-Exactive dataset. (c) Testing on a dataset of standard proteins. All b ions are disabled while searching, and a conventional target-decoy approach with reversed protein sequences as decoys is used. The legend "+ FMM" means the finite mixture model was used to estimate the correct and incorrect score distributions. And "Decoy: [1, 30]" means the decoy is generated by adding a random mass ranging from 1 to 30 Da to each theoretical y ion.

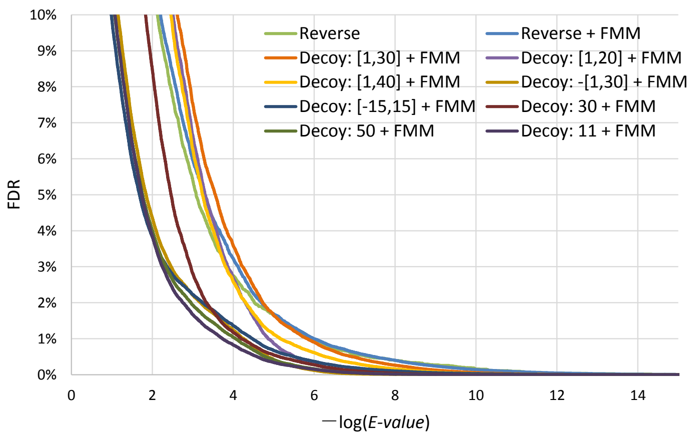

Figure S-4. Looking for an optimal spectrum-based decoy method on the Orbitrap Velos dataset. “Decoy: 11” means the spectrum-based decoy is generated by adding 11 Da to each theoretical y ion, other legend descriptions are similar to **Figure S-3**.

Best parameter for the Y_1 ion filtration

The trimannosyl core is the stable structure in N-glycans, and it is feasible to use this information to filter out unreliable Y_1 ions. In pGlyco, there should be at least 3 matched trimannosyl core ions or a (Y_1, Y_1*) ion pair with the same charge state for a reliable Y_1 ion. This parameter should be verified for its optimality. The verification could be done based on the 765 manually checked GPSMs, as shown in **Table S-3**. Under the default Y_1 filtration condition of pGlyco, the sensitivity and accuracy were 80.0% and 99.8% at 1% glycan FDR (the peptide FDR of MS3 identification was 1%). 20.0% true positive GPSMs did not get a high score for filtration. When using the Y_1/Y_1* ion pair alone, the sensitivity decreased to 53.5%. And when using "core ≥ 3" alone, the sensitivity and accuracy were quite acceptable, so it was not bad to use "core ≥ 3" only for the Y_1 ion filtration as described in previous work [9]. The accuracy increased from 84.7% to 90.4% when using the parameter "core ≥ 3 or Y_1/Y_1*", as compared to the parameter "core ≥ 3". When increasing the number of matched trimannosyl core ions to 4, 5 or 6, the sensitivity dropped down, although the accuracy was very high even without FDR cutoff. To balance the sensitivity and accuracy, pGlyco used the condition "core ≥ 3 or Y_1/Y_1*" to filter Y_1 ions. The parameter "core ≥ 1 (or 2)" was not tested in **Table S-3**, because it was difficult to judge if the GPSM is correct with only one or two trimannosyl core ions matched.

Table S-3. The sensitivity and accuracy of the Y_1 ion filtration under different parameters. The parameter "core ≥ x" means the Y_1 ion is filtered by at least x trimannosyl core ions. "Sensitivity" or "accuracy" means the sensitivity or accuracy is tested after the filtration with the glycan FDR ≤ 1% by pGlyco. And "sensitivity (no FDR)" or "accuracy (no FDR)" means the sensitivity or accuracy is tested without the filtration of glycan FDR ≤ 1%. The parameter "core ≥ 1 (or 2)" is not tested here, because it is difficult to judge if a GPSM is correct with only one or two trimannosyl core ions matched.

parameters	core ≥ 3 or Y_1/Y_1*	Y_1/Y_1*	core ≥ 3	core ≥ 4	core ≥ 5	core ≥ 6
sensitivity	80.0%	53.5%	79.3%	74.1%	59.5%	22.5%
sensitivity (no FDR)	100%	61.1%	98.0%	81.5%	59.5%	41.5%
accuracy	99.8%	100%	99%	99.8%	99.8%	100%
accuracy (no FDR)	90.4%	94.0%	84.7%	98.1%	99.8%	100%

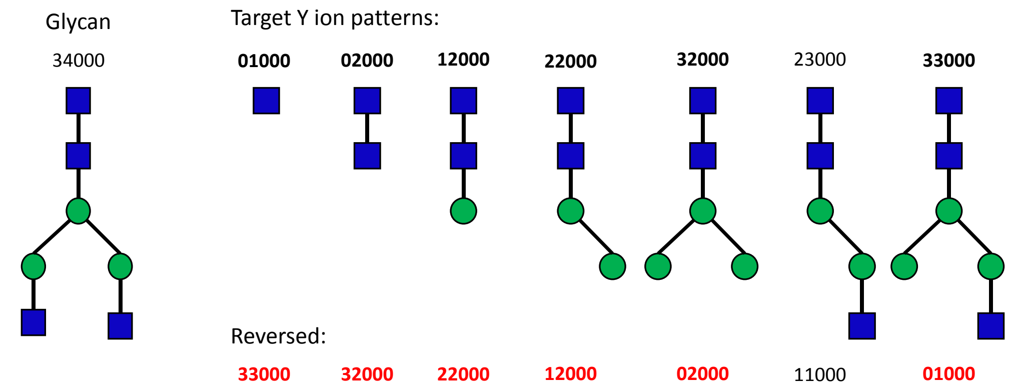

Figure S-5. Y ion patterns and their reverse of the glycan (3, 4, 0, 0, 0) (we defined reversed Y ion composition = glycan composition – Y ion composition). We omit the commas in the representation of the glycan and its Y ions in this figure for simplicity. A reversed Y ion is calculated as the parent glycan minus the target Y ion. For example, the reversal of the target Y ion 12000 is 22000 (3400 − 12000 = 22000). 6 Y ion compositions (the red font) are shared by the target and its reversal, resulting in a difficulty in distinguishing them.

MS3 fragmentation modes — HCD or CID

We compared different modes, HCD and CID, for MS3 fragmentation, and results were shown in **Figure S-6** and **Figure S-7.** The parent ion of the CID MS3 spectrum of the peptide "NEEYJ[+HexNAc]K" was not completely fragmented, leaving highly intense Y_0, Y_1* and Y_1−H_2O ions in (**Figure S-6a**). But this peptide was well fragmented by HCD (**Figure S-6b**), in which the intensities of Y_0, Y_1* and Y_1−H_2O were much lower. Furthermore, HCD fragmentation has overcome the low-mass cutoff problem [10], so it provides more complete b, y ions for the peptide identification. Although sometimes the parent ion of MS3 was not well fragmented in HCD, leaving highly intense Y_1 and Y_1* ions, such as the peptide "LVPVPITJ[+HexNAc]ATLDR", as shown in **Figure S-6d**, the sequence coverage was still better than CID-MS3 in **Figure S-6c**. The global comparison between these two MS3 fragmentations was shown in **Figure S-7**. Therefore, from our analysis, the HCD fragmentation for MS3 analysis was preferred. But there were probably some bugs in our Orbitrap Fusion, leading to lower throughput of HCD MS3.

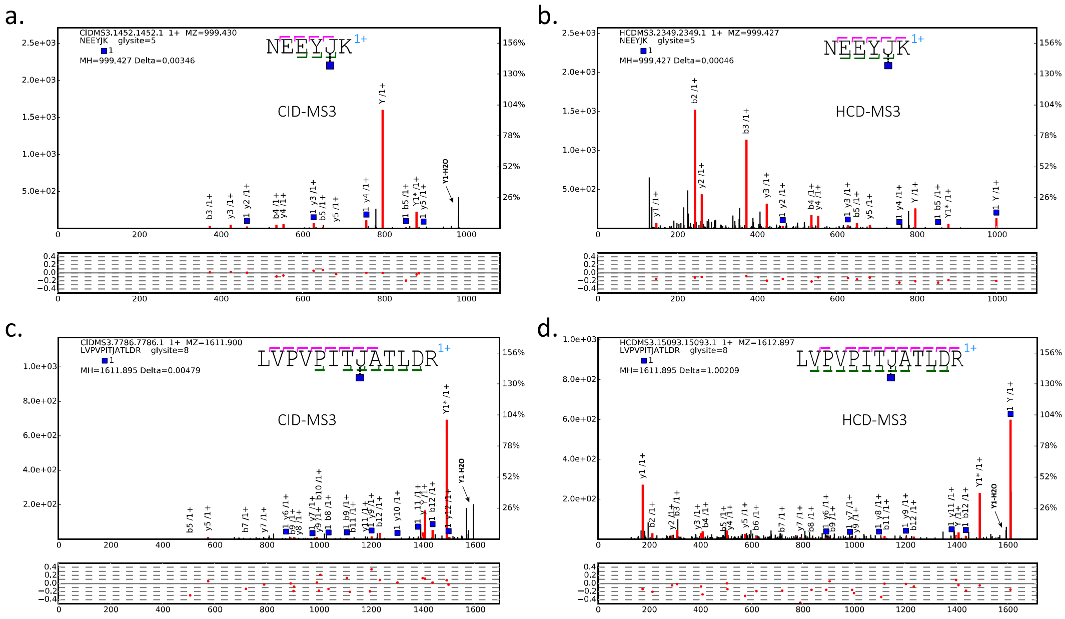

Figure S-6. CID and HCD fragmentation for MS3 of the peptide "NEEYJ[+HexNAc]K" and the peptide "LVPVPITJ[+HexNAc]ATLDR". (**a**) The CID MS3 spectrum of "NEEYJ[+HexNAc]K". (**b**) The HCD MS3 spectrum of "NEEYJ[+HexNAc]K". (**c**) CID MS3 of "LVPVPITJ[+HexNAc]ATLDR". (**d**) HCD MS3 of "LVPVPITJ[+HexNAc]ATLDR". Both spectra in (**a**) and (**b**), or both spectra in (**c**) and (**d**) have the same precursor charge states and similar basepeak intensities, which makes them comparable.

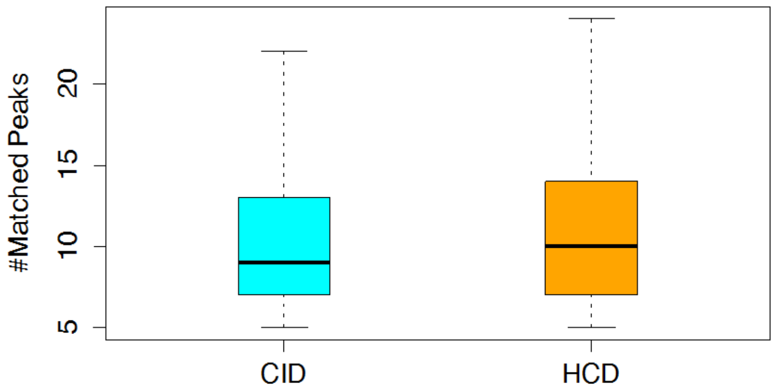

Figure S-7. The boxplot of the number of peaks matched in CID and HCD spectra for MS3 analysis. HCD could provide more b/y ions for the identification of peptide backbones. It is mainly because of the overcoming of 28% cutoff and the more complete fragmentation of HCD.

References

1. Lynn, K.S., et al., *MAGIC: an automated N-linked glycoprotein identification tool using a Y1-ion pattern matching algorithm and in silico MS(2) approach.* Anal Chem, 2015. **87**(4): p. 2466-73.
2. Elias, J.E. and S.P. Gygi, *Target-decoy search strategy for increased confidence in large-scale protein identifications by mass spectrometry.* Nat Methods, 2007. **4**(3): p. 207-14.
3. Schwarz, G., *Estimating Dimension of a Model.* Ann Stat, 1978. **6**(2): p. 461-4.
4. Efron, B. and R. Tibshirani, *Empirical bayes methods and false discovery rates for microarrays.* Genet Epidemiol, 2002. **23**(1): p. 70-86.
5. Kall, L., et al., *Posterior error probabilities and false discovery rates: Two sides of the same coin.* J Proteome Res, 2008. **7**(1): p. 40-4.
6. Michalski, A., et al., *Mass spectrometry-based proteomics using Q Exactive, a high-performance benchtop quadrupole Orbitrap mass spectrometer.* Mol Cell Proteomics, 2011. **10**(9): p. M111 011015.
7. Chi, H., et al., *pNovo: de novo peptide sequencing and identification using HCD spectra.* J Proteome Res, 2010. **9**(5): p. 2713-24.
8. Chi, H., et al., *pNovo+: de novo peptide sequencing using complementary HCD and ETD tandem mass spectra.* J Proteome Res, 2013. **12**(2): p. 615-25.
9. Cheng, K., et al., *Large-scale characterization of intact N-glycopeptides using an automated glycoproteomic method.* J Proteomics, 2014. **110**(14): p. 145-54.
10. Jedrychowski, M.P., et al., *Evaluation of HCD- and CID-type fragmentation within their respective detection platforms for murine phosphoproteomics.* Mol Cell Proteomics, 2011. **10**(12): p. M111 009910.
